# Acupuncture for sequelae of Bell's palsy: a randomized controlled trial protocol

**DOI:** 10.1186/1745-6215-12-71

**Published:** 2011-03-09

**Authors:** Hyo-Jung Kwon, Jong-In Kim, Myeong Soo Lee, Jun-Yong Choi, Sungkeel Kang, Jie-Yoon Chung, Young-Jin Kim, Seung-Hoon Lee, Sanghoon Lee, Dongwoo Nam, Yong-Suk Kim, Jae-Dong Lee, Do-Young Choi

**Affiliations:** 1 Facial Palsy Centre, Department of Acupuncture & Moxibustion, College of Korean Medicine, Kyung Hee University, Seoul, Republic of Korea; 2 Brain Disease Research Center, Korea Institute of Oriental Medicine, Daejeon, Republic of Korea; 3 Department of Internal Medicine, School of Korean Medicine, Pusan National University, Yangsan, Republic of Korea

## Abstract

**Objective:**

Incomplete recovery from facial palsy has a long-term impact on the quality of life, and medical options for the sequelae of Bell's palsy are limited. Invasive treatments and physiotherapy have been employed to relieve symptoms, but there is limited clinical evidence for their effectiveness. Acupuncture is widely used on Bell's palsy patients in East Asia, but there is insufficient evidence for its effectiveness on Bell's palsy sequelae. The objective is to evaluate the efficacy and safety of acupuncture in patients with sequelae of Bell's palsy.

**Method/Design:**

This study consists of a randomized controlled trial with two parallel arms: an acupuncture group and a waitlist group. The acupuncture group will receive acupuncture treatment three times per week for a total of 24 sessions over 8 weeks. Participants in the waitlist group will not receive any acupuncture treatments during this 8 week period, but they will participate in the evaluations of symptoms at the start of the study, at 5 weeks and at 8 weeks after randomization, at which point the same treatment as the acupuncture group will be provided. The primary outcome will be analyzed by the change in the Facial Disability Index (FDI) from baseline to week eight. The secondary outcome measures will include FDI from baseline to week five, House-Brackmann Grade, lip mobility, and stiffness scales.

**Trial registration:**

Current Controlled-Trials ISRCTN43104115; registration date: 06 July 2010; the date of the first patient's randomization: 04 August 2010

## Background

Bell's palsy is an acute, idiopathic, unilateral paralysis of the face. Its cause is unknown, but mounting evidence suggests that reactivated herpes viruses from cranial nerve ganglia play a key role in the development of this condition; its pattern is consistent with that of peripheral neural dysfunction [1,2]. Inflammation of the facial nerve initially results in reversible neuropraxia and Wallerian degeneration ultimately ensues [1].

The incidence of Bell's palsy is approximately 30/100,000 people per year [3]. The prognosis is good, and approximately 70% of patients recover completely within 6 months without treatment. However, 30% of Bell's palsy patients have sequelae, such as residual paresis (29%), contracture (17%), and facial spasm or synkinesis (16%) [3]. The incomplete recovery of facial symmetry can have a long-term impact on the quality of life, such as difficulty with drinking, eating and speaking, as well as psychosocial problems [4]. The symmetry of the face is a determinant of facial charm and influences interpersonal attraction [5].

Medical options for the sequelae of Bell's palsy are limited to invasive treatments, such as injection of Botulinum toxin A and surgical reconstruction. Patients may be referred to physiotherapists as well, although there is no evidence favoring one intervention over another [5-8]. According to a 2009 study, the effect of steroids on acute Bell's palsy within 72 hours of the onset of symptoms is clinically effective, but steroids are not used on the sequelae of Bell's palsy [9,10].

Acupuncture is known to be a safe treatment and is used for a wide range of symptoms associated with Bell's palsy [11]. In a 2007 systematic review, the effect of acupuncture on Bell's palsy was inconclusive [12]. This stems from limited clinical studies on acupuncture for Bell's palsy and various errors in the design of some of those studies. To overcome such problems, a large-scale clinical trial investigating the effect of acupuncture on Bell's palsy is under way [13]. Here, our aim is to evaluate the safety and efficacy of acupuncture on the sequelae of Bell's palsy.

## Method/Design

### Objective

The primary objective of this study is to investigate the efficacy and safety of acupuncture in patients with sequelae of Bell's palsy.

### Primary outcome

The change in the Facial Disability Index (FDI) social [14] score after completing eight weeks of acupuncture treatment will be compared to the score prior to treatment. This scoring system consists of two domains; physical score and social score, and has been investigated for the reliability and validity [14]. FDI-social score evaluations will be performed on the first visit, the fifth- and eighth-week visits. Participants will answer five multiple choice questions related to problems associated with facial muscle function during the previous month.

### Secondary outcome

Secondary outcome measures will include FDI-social score from baseline to week five, FDI-physical score, House-Brackmann (H-B) Grade [15], lip mobility (lip-length and snout indices) [16], and stiffness scales [17]. The H-B grade has been tested for its reliability [18] and lip-length and snout indices showed a high correlation with H-B Grade [19]. Stiffness scale is a simple five-point scale for facial stiffness (1 = no stiffness, 5 = very stiff) [17].

Unfortunately, there has been no validation study of Korean version of each of the primary and secondary outcomes. In our study, two professional translators made each outcome measurement questionnaire in Korean, which has been used in our clinical facial palsy center for evaluating sequelae of facial palsy. So we decided that these questionnaires are readable and suitable to our study.

### Design

**Figure 1:**
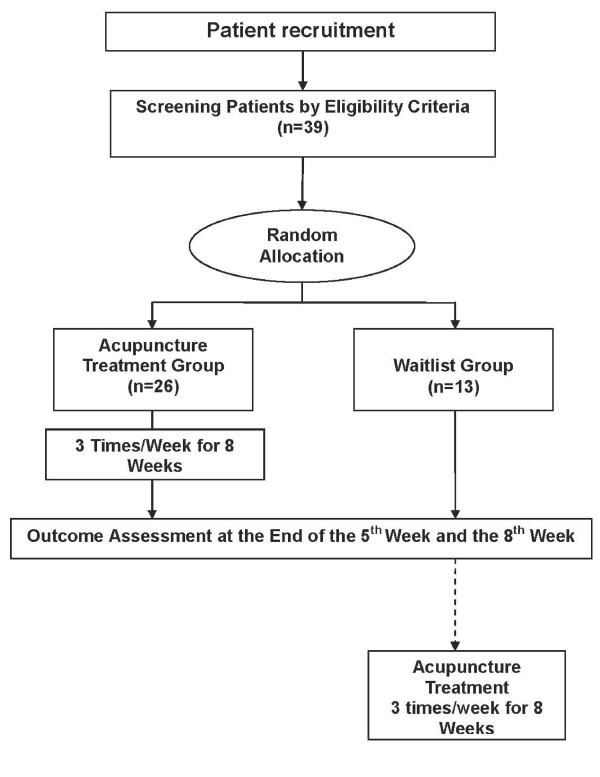
**Chronological Flow Chart of the Study Design**.

A randomized, assessor-blinded, waitlist controlled trial will be conducted at the Acupuncture and Moxibustion Department of Kyung Hee University's Oriental Medical Hospital in Seoul. (Figure 1) The study includes a screening period before randomization and a treatment period of 8 weeks (3 sessions per week). After screening for inclusion/exclusion criteria, subjects will be randomized to either the acupuncture group or the waitlist group, in accordance with a computer-generated random number list that is hidden until the start of treatment. No-acupuncture waitlist control is used as a control because sham acupuncture cannot be substituted for a physiologically inert placebo [20].

### Eligibility

#### Inclusion criteria

To be included, patients have to be diagnosed with Bell's palsy at least six months prior to screening. Patients must score less than 70 on the physical aspect of the FDI, less than 80 on the social aspect and provide written informed consent. Participants who are 18 to 65 years old will be recruited using local newspaper advertisements and posted notices.

#### Exclusion criteria

Participants will be excluded if they suffer from serious medical conditions, such as uncontrolled hypertension, diabetes mellitus requiring insulin injection, past or current malignant tumors, severe dyslipidemia, liver or kidney dysfunction, anemia, active pulmonary tuberculosis or other infectious or systemic diseases that are inappropriate for treatment with acupuncture.

Eligible participants will be excluded if they have complicated pathophysiologic conditions of secondary facial palsy from infection, multiple neuritis, tumors invading the temporal bone, brain contusion or stroke. Patients with Ramsay-Hunt Syndrome [21] will also be excluded from the study, as well as those with bilateral or recurrent facial palsy. To avoid confounding effects that can influence patients' outcome measures, the oral administration of steroids or antiviral drugs (acyclovir, valaciclovir, famciclovir, or ganciclovir), a surgical history for facial palsy, such as facial nerve decompression, reconstruction of the facial nerve or muscle, and history of acupuncture, moxibustion, vesiculation and massage therapy within three months will also result in exclusion from the study. With regard to safety and compliance issues, patients with other neurological illnesses, neuropsychiatric diseases, present or planned pregnancy, current lactation, or scars on the treatment area will also be excluded.

Over-the-counter (OTC) drugs for common colds, dyspepsia, headaches, etc. will be allowed. However OTC drugs of unknown components or indicated for Bell's palsy related symptoms will not be allowed. All patients will be instructed to disclose all medication use prior to enrollment. Records on the drugs taken by each patient will be obtained at every visit, and patients will be requested to inform us of any change to their medication or supplement regimen. Additional acupuncture treatments, herbal medicines, or medical interventions elsewhere will not be allowed throughout the study.

### Acupuncture treatment protocol

#### Acupuncture intervention

Acupuncture will be performed by specialists in oriental medicine. Participants will be randomly assigned to the acupuncture treatment group or the waitlist group. The acupuncture group will receive acupuncture treatments 3 times/week for a total of 24 sessions over 8 weeks, following the details of the STandards for Reporting Interventions in Clinical Trials of Acupuncture (STRICTA) 2010 checklist as shown in Table 1. In the acupuncture treatment group, 18 acupuncture points [22] (ST4, ST6 on the unaffected side, ST1, EX-HN4, TE23, LI20 on the affected side, TE17, ST9, LI10, LI4, ST36 and GB34 on both sides) will be used. These selected acupuncture points were based on acupuncture specialist forums and the textbook of acupuncture in Korea [23].

**Table 1:** Details of the proposed acupuncture intervention according to the STRICTA 2010 Checklist

Acupuncture rationale	Style of acupuncture	Traditional Korean Medicine
	
	Rationale for treatment	Acupuncture has been historically used to treat facial palsy. Additionally, it is known to be a safe treatment used in a wide range of symptoms caused by Bell's palsy [11].
	
	Extent to which treatment varied	The subjects of the acupuncture group all receive the same treatment
**Details of needling**	Number of needle insertions per subject per session	18
	
	Names of the insertion points (uni/bilateral)	ST4, ST6, (unilateral, unaffected side) ST1, EX-HN4, TE23, LI20 (unilateral, affected side) TE17, ST9, LI10, LI4, ST36 and GB34 (bilateral)
	
	Depth of insertion	5-30 mm (exact depth shown in Table 2)
	
	Response sought	De-qi
	
	Needle stimulation	Manual
	
	Needle retention time	10 minutes
	
	Needle type	0.20 mm (diameter) × 30 mm (length) disposal needle (Dongbang Acupuncture Inc, Boryeong, Korea)

**Treatment regimen**	Number of treatment sessions	24
	
	Frequency and duration of treatment sessions	3 sessions/week for 8 weeks

**Other components of treatment**	Details of other interventions administered to the acupuncture group	No other interventions are done
	
	Setting and context of treatment	All subjects are informed that they will receive acupuncture treatment, which can potentially reduce the sequelae of Bell's palsy symptoms; however, the control group would have to complete the three evaluations during the first 8 weeks before receiving the same treatment as the acupuncture group.

**Practitioner background**	Description of participating acupuncturists	Specialists in Oriental Medicine with at least 3 years of practice in acupuncture

**Control or comparator interventions**	Rationale for the control or comparator in the context of the research question	No-acupuncture waitlist control is used as a control because sham acupuncture cannot be a substituted for a physiologically inert placebo [20]
	
	Precise description of the control or comparator	The control group forms a waitlist and completes the evaluations during the first 8 weeks after randomization before receiving the same treatment as the acupuncture group.

A 0.20-mm (diameter) × 30-mm (length) disposal needle (Dongbang Acupuncture Inc, Boryeong, Korea) (Table 1) will be inserted into each acupuncture point to a depth of 5-30 mm, depending on the points selected. All participating acupuncture doctors will use de-qi sensation techniques to manually manipulate and maintain the needles for a 10-minute period with manipulation at the start and end of the 10-minute period. Additional interventions, i.e., infrared irradiation or electronic stimulation, will not be allowed during the acupuncture treatment. The ST4 and ST6 needles will penetrate to a depth of 20 to 30 mm using an oblique angle of insertion toward each other's tips. The ST1 and EX-HN4 needles will penetrate to a depth of 5 to 10 mm at an oblique angle toward the eye, using caution so as not to damage the conjunctiva when applying the ST1 needle. The TE23 needle will penetrate to a depth of 20 to 30 mm at an oblique angle toward the ear. The LI20 needle will be inserted to a depth of 20 to 30 mm at an oblique angle toward the root of the nose. The TE17, ST9, LI10, LI4, ST36 and GB34, needles will be inserted perpendicular to the skin to a depth of 20 to 30 mm. (Table 2) These manipulations will be repeated after 10 minutes. Needling will be carried out with participants in the supine position, and needle sites will be swabbed with 2% boric acid before insertion. Upon withdrawal of the needle, dry sterilized cotton balls will be firmly applied to the insertion points.

**Table 2:** Acupuncture Points and Needling Procedure

Acupuncture Point	Direction	Depth (mm)
ST4 (unaffected side)	Transversely, toward ST6	20-30

ST6 (unaffected side)	Transversely, toward ST4	20-30

ST1 (affected side)	Transversely, toward the eye	5-10

EX-HN4 (affected side)	Transversely, toward the eye	5-10

TE23 (affected side)	Transversely, toward the ear	20-30

LI20 (affected side)	Obliquely along nasolabial sulcus toward the root of nose	20-30

TE17 (both sides)	Perpendicular to the skin	20-30

ST9 (both sides)	Perpendicular to the skin	20-30

LI10 (both sides)	Perpendicular to the skin	20-30

LI4 (both sides)	Perpendicular to the skin	20-30

ST36 (both sides)	Perpendicular to the skin	20-30

GB34 (both sides)	Perpendicular to the skin	20-30

### Outcome measures

After screening, the participants will answer the FDI questionnaire three more times: at the first visit, at five weeks, and at eight weeks after the final treatment. The assessment of H-B Grade, lip mobility, and stiffness will be conducted at each time point.

The expectations of the participants will be recorded using the credibility and expectancy questionnaire [24] to determine whether expectations affected outcomes.

Any adverse events as a result of acupuncture will be monitored and recorded. At the end of the trial, the participants in the acupuncture group will be asked if they are satisfied with the outcome.

Because the knowledge of the group assignment could modify outcome assessment, we will ensure that the independent assessor is blinded.

### Statistical methods

#### Analysis

Analysis will be performed for an intention-to-treat population consisting of all randomized participants regardless of their actual treatment received. Any missing data will be replaced with ones by a multiple imputation technique [25]. All data will be analyzed descriptively. Of the primary and secondary outcome measurements, variables of FDI, H-B grade and stiffness scale are discrete ordinal variables whereas lip mobility is recorded as a continuous variable. For primary and secondary outcome measurements, the mean differences from the baseline values to the end of treatment will be compared between the two groups using the two-sample t-test if the data are distributed normally. In case of non-normally distributed data, Wilcoxon rank-sum test will be performed. If baseline values of outcome measurements are significantly different between groups, analysis of co-variance (ANCOVA) will be performed using any imbalanced variables as covariates and assigned group as fixed factor.

All adverse events reported during the study will be included in the clinical report, and the prevalence of adverse events will then be calculated. The percentage of subjects with adverse events in each group will be calculated and compared using the chi-squared test or Fisher's exact test.

Statistical analyses will be performed using the SAS statistical package program (ver. 9.1.3), and the level of significance will be established at α = 0.05.

#### Sample size

Sample size was calculated from the result of a mime therapy trial conducted on patients with sequelae of Bell's palsy of which design was randomized parallel waitlist controlled trial [17]. We assumed that the effect size of our invasive acupuncture therapy will be at least equal to this non-invasive mime therapy. Also we decided the allocation ratio to be 2:1 with the consideration that fewer patients should be allocated to the waitlist group.

In the mime trial, the mean difference of follow-up FDI-social score between groups was 14.5 and pooled standard deviation was 14.5 within each group [17]. The alpha value and power were 0.05 and 0.8 respectively. From these values we calculated sample size for independent two sample t-test (one-tailed) that is 26 for acupuncture group and 13 for waitlist group with consideration of 20% drop-out. And this assumed mean difference of FDI-social score between groups is considered to be sufficiently important in an expert discussion consisting of clinicians who work in Bell's palsy clinics for more than 10 years.

#### Data handling

Investigators will enter the information required by the protocol into the case report forms. Unclear entries and omissions will be entered on data query forms, which will be returned to the investigational site for resolution. The data will be summarized according to demographic baseline characteristics, effectiveness and safety observations.

#### Data and safety monitoring

Regular monitoring will be conducted at the investigational site for quality control. Additionally, investigators convene to discuss practical issues that may be encountered, such as adjusting recruitment capacity, dealing with serious adverse events, revising the protocol, and other important issues that may be raised by investigators and participants. The assessment of safety will primarily be based on the frequency of adverse events, which will include all serious adverse events. Information regarding adverse events will be summarized by presenting the number and percentage of the participants who experienced them. The data will also be categorized according to the affected body region. All of the other information collected (e.g., severity or relation to acupuncture treatments) will be listed appropriately.

#### Randomization and allocation

Computerized randomization will be performed by an outside researcher who will not be in direct contact with the participants. The assessor will be blinded to the group allocation. The investigator performing the acupuncture intervention cannot be intrinsically blinded, but will not be allowed to communicate with the participants or the assessor about the treatment procedures and outcomes. A sealed envelope containing allocation sequence number for each patient will be opened right after each patient meets eligibility criteria and informed consent is made. If any error or disclosure with regard to randomization occurs, a new randomization sequence will be generated starting from the problematic serial number and applied to the patients from then on.

Ultimately, 39 participants will be enrolled in the study.

A balanced block randomization will be used to assign 26 participants to the acupuncture group and 13 to the waitlist group; the exact block size will be concealed from any personnel who will be in direct contact with patients. Following the baseline assessment, eligible participants will be assigned to one of two groups: a group receiving acupuncture or a control waitlist group. All subjects will be informed that there is a possibility they will be allocated to the waitlist control group and in that case, they should wait 8 weeks before receiving acupuncture treatments during 8 weeks. To eliminate observation bias, the independent assessor will be blinded prior to the analysis of the data.

### Ethics

Written informed consent will be obtained from each participant. This study is approved by the Institutional Review Board of Kyung Hee University's Oriental Medical Hospital.

## Abbreviations

ANCOVA: analysis of co-variance (ANCOVA); FDI: Facial Disability Index; H-B: House-Brackmann.

## Competing interests

The authors declare that they have no competing interests.

## Authors' contributions

HJK and JIK drafted the protocol and HJK wrote the final manuscript. JYC was responsible for the statistical design of the trial and wrote a part of the statistical methods, data handling and monitoring sections. MSL, JYC, YJC, SHL, SL, DWN, JDL, and DYC contributed to the study design and made critical revisions. SKK and YSK edited the technical part of the study. All authors read and approved the final manuscript. JIK is the principal investigator of this study.
